# Supplementary material for: Causal Relationship between Aging and Anorexia Nervosa: A White-Matter-Microstructure-Mediated Mendelian Randomization Analysis
Source: Biomedicines. 2024 Aug 16;12(8):1874. doi: 10.3390/biomedicines12081874 (PMC11351342; doi:10.3390/biomedicines12081874)
Supplement: Supplementary file 1 [file biomedicines-12-01874-s001.zip › Supplementary Figure/Figure S4/Singe SNP plot of MR analysis for TL to ubm-b-1902.pdf]

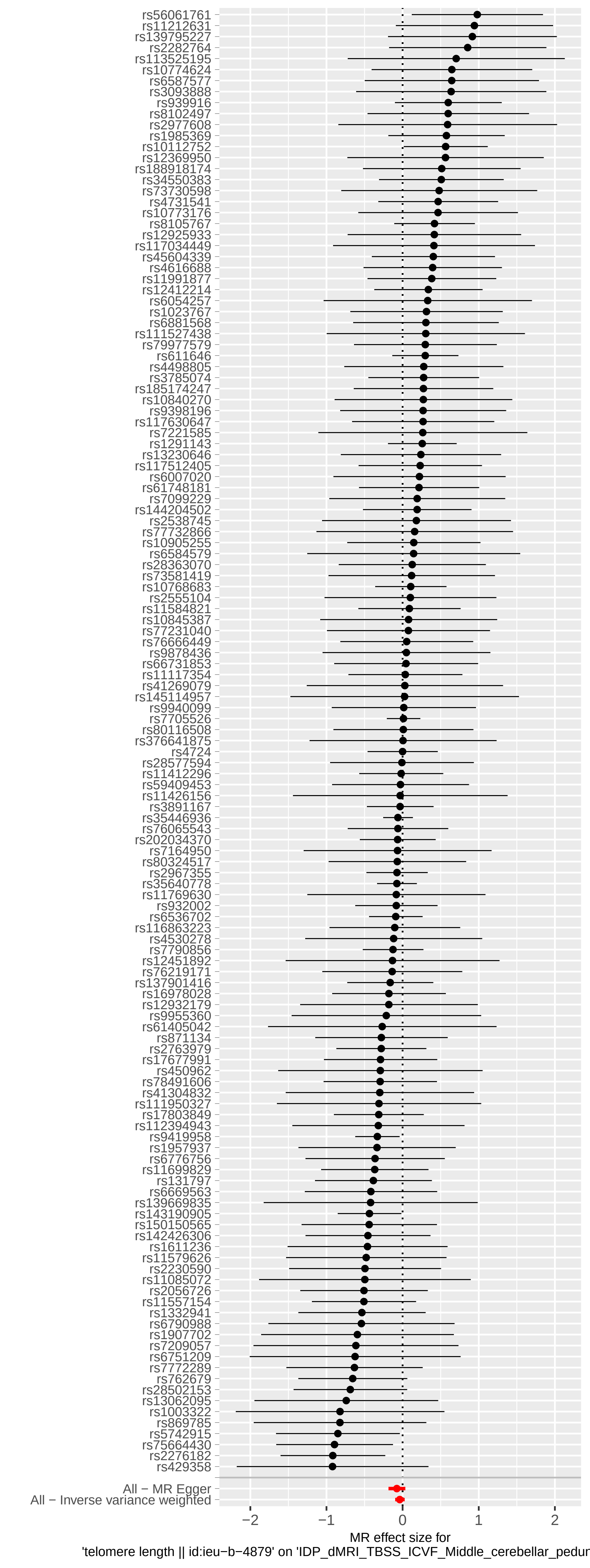
rs56061761
rs11212631
rs139795227
rs2282764
rs113525195
rs10774624
rs6587577
rs3093888
rs939916
rs8102497
rs2977608
rs1985369
rs10112752
rs12369950
rs188918174
rs34550383
rs73730598
rs4731541
rs10773176
rs8105767
rs12925933
rs117034449
rs45604339
rs4616688
rs11991877
rs12412214
rs6054257
rs1023767
rs6881568
rs111527438
rs79977579
rs611646
rs4498805
rs3785074
rs185174247
rs10840270
rs9398196
rs117630647
rs7221585
rs1291143
rs13230646
rs117512405
rs6007020
rs61748181
rs7099229
rs144204502
rs2538745
rs77732866
rs10905255
rs6584579
rs28363070
rs73581419
rs10768683
rs2555104
rs11584821
rs10845387
rs77231040
rs76666449
rs9878436
rs66731853
rs11117354
rs41269079
rs145114957
rs9940099
rs7705526
rs80116508
rs376641875
rs4724
rs28577594
rs11412296
rs59409453
rs11426156
rs3891167
rs35446936
rs76065543
rs202034370
rs7164950
rs80324517
rs2967355
rs35640778
rs11769630
rs932002
rs6536702
rs116863223
rs4530278
rs7790856
rs12451892
rs76219171
rs137901416
rs16978028
rs12932179
rs9955360
rs61405042
rs871134
rs2763979
rs17677991
rs450962
rs78491606
rs41304832
rs111950327
rs17803849
rs112394943
rs9419958
rs1957937
rs6776756
rs11699829
rs131797
rs6669563
rs139669835
rs143190905
rs150150565
rs142426306
rs1611236
rs11579626
rs2230590
rs11085072
rs2056726
rs11557154
rs1332941
rs6790988
rs1907702
rs7209057
rs6751209
rs7772289
rs762679
rs28502153
rs13062095
rs1003322
rs869785
rs5742915
rs75664430
rs2276182
rs429358
All − MR Egger
All − Inverse variance weighted
−2
−1
0
1
2
MR effect size for
'telomere length || id:ieu−b−4879' on 'IDP_dMRI_TBSS_ICVF_Middle_cerebellar_pedur
